# Reliability and Validity Assessment of the Observation of Human-Animal Interaction for Research (OHAIRE) Behavior Coding Tool

**DOI:** 10.3389/fvets.2018.00268

**Published:** 2018-11-08

**Authors:** Noémie A. Guérin, Robin L. Gabriels, Monique M. Germone, Sabrina E. B. Schuck, Anne Traynor, Katherine M. Thomas, Samantha J. McKenzie, Virginia Slaughter, Marguerite E. O'Haire

**Affiliations:** ^1^Department of Comparative Pathobiology, Center for the Human-Animal Bond, College of Veterinary Medicine, Purdue University, West Lafayette, IN, United States; ^2^Department of Psychiatry, School of Medicine, University of Colorado, Denver, CO, United States; ^3^Child Development Center, Pediatrics School of Medicine, University of California, Irvine, Irvine, CA, United States; ^4^Department of Educational Studies, College of Education, Purdue University, West Lafayette, IN, United States; ^5^Department of Psychological Sciences, College of Health and Human Sciences, Purdue University, West Lafayette, IN, United States; ^6^Institute for Teaching and Learning Innovation, The University of Queensland, Brisbane, QLD, Australia; ^7^School of Psychology, The University of Queensland, Brisbane, QLD, Australia

**Keywords:** human-animal interaction, animal-assisted intervention, social behaviors, behavior coding, interval coding, psychometrics

## Abstract

The Observation of Human-Animal Interaction for Research (OHAIRE) is a coding tool developed to capture the behavior of children when interacting with social partners and animals in naturalistic settings. The OHAIRE behavioral categories of focus are emotional displays, social communication behaviors toward adults and peers, behaviors directed toward animals or experimental control objects, and interfering behaviors. To date, the OHAIRE has been used by 14 coders to code 2,732 min of video across four studies with a total of 201 participants ages 5 to 18 years (*M* = 10.1, *SD* = 2.5). Studies involved animal-assisted intervention with three species (i.e., dogs, horses, and guinea pigs) and three populations (i.e., autism spectrum disorder, attention-deficit hyperactivity disorder, and typically developing children) in a school, a therapeutic horseback riding program, a group therapy program, and the hospital setting. We explored the psychometric properties of the OHAIRE through analyses of its inter-rater reliability, intra-rater reliability, convergent and divergent validity, and internal structure, using data from these four human-animal interaction studies. The average inter-rater reliability was excellent (kappa = 0.81), with good reliability in most of the behavioral categories coded. Intra-rater reliability was consistently excellent (0.87 ≤ kappa ≤0.96). Internal structure analyses with Cronbach's alpha supported the exploratory use of subscales to measure social communication behaviors toward peers (α = 0.638) and adults (α = 0.605), and interactions experimental control objects (α = 0.589), and the use of a subscale to measure interactions with animals (α = 0.773). Correlation analyses with multiple questionnaires showed a convergence between positive emotional display and social behaviors as assessed by the OHAIRE and social skills as assessed by the Social Skills Rating System (SSRS) and the Social Communication Questionnaires (SCQ). Little concordance was found between the OHAIRE and the Social Responsiveness Scale (SRS) or the Aberrant Behavior Checklist-Community (ABC). The OHAIRE shows promise for wider use in the field of Human-Animal Interaction, with a need for generalization across more settings and ages.

## Introduction

### Background

The notion that animals can affect people's lives and behaviors in many positive ways is investigated in a field of research known as Human-Animal Interaction (HAI). As a relatively recent and interdisciplinary field, HAI is often criticized for its lack of methodological rigor (1, 2). Common HAI research critiques target weak study design, small sample sizes, and the inappropriate use of assessment tools, which limits the field's ability to develop an evidence base for animal-assisted intervention (AAI). Assessment in HAI research has relied heavily on questionnaire data and there has been a call to use more physiological measures and behavioral observation.

Physiological measures and behavioral observation are considered more objective than questionnaires, because they quantify observable physical phenomena rather than mental experiences as reported by a study participant's or caregiver's perceptions. Yet, while the instruments used to collect physiological data rely on direct physical measures (e.g., heart beats per minute) and assays (e.g., salivary cortisol), thus reducing the influence of human error, the quantification of behavior still requires the direct involvement of a human observer. To assess behavior, a human observer typically watches study participants directly or via a video recording and assigns numerical values to the participants' behaviors based on precise behavior definitions. From the combination of such behavior definitions with sampling and scoring procedures, researchers can develop standardized coding schemes or systems.

Standardized assessment tools are critical to building an empirical base for the HAI field by yielding results that are replicable and comparable across studies. Ultimately, the use of standardized assessment facilitates conducting meta-analyses, which summarize the empirical evidence available in the current literature on a specific topic (3, 4). While the use of standardized behavior observation schemes is common practice in the field of psychology, we are not aware of a published, validated tool that incorporates behaviors relevant to the study of HAI, that is, behaviors directed toward animals.

To address the need for a standardized human behavior coding tool adapted to HAI research, the Observation of Human-Animal Interaction for Research (OHAIRE) was developed. The OHAIRE is a behavior coding tool developed to capture the behavior of humans when interacting with social partners and animals in naturalistic settings. Here, we define naturalistic settings as any setting where participants are not asked to perform specific tasks and are free to interact with each other and with any animal present. We do not recommend the use of the OHAIRE in settings where behaviors are heavily directed (i.e., with a detailed agenda), as we are seeking to capture natural variations in willful social interactions across conditions. Behaviors captured in the OHAIRE coding tools were selected based on common research questions, commonly evaluated outcomes, and the main theories of focus in HAI research.

Four of the main theories applied in HAI research are grounded in evolutionary biology and social psychology (5). The two main evolutionary theories informing HAI research are Biophilia and Neoteny. Biophilia postulates that humans are inherently drawn to the living beings around them (6), while Neoteny refers to the presence of juvenile characteristics (e.g., large eye to head ratio, play behaviors) in adult domesticated animals, encouraging social and nurturing behaviors from humans (7). Both theories hypothesize that human beings naturally display a certain level of behavioral attention (e.g., social and nurturing) toward animals. This direct display of attention sometimes encourages social behaviors directed toward animals and, leads to the creation of a human-animal bond.

The human-animal bond has been hypothesized to fit within the psychological theories of social support and attachment (5). In the social support theory framework (8), interactions with companion animals may reduce loneliness and be a source of social support for humans, as well as encourage social interactions with other humans, while attachment theory (9) applied to HAI suggests that human beings may develop attachment bonds to animals, providing emotional safety. Taken together, these theories have shaped the research questions and outcomes evaluated in socio-emotional HAI research.

To accommodate these common research questions and theories, the behavioral categories captured in the OHAIRE include social interactions, interactions with animals and control objects, emotional display, and interfering behaviors. Specific behaviors are captured to address prevalent theories, including attention to humans and animals (Biophilia), prosocial or caring behaviors (Neoteny), social interactions (social support theory), and human-animal bond (attachment theory). The OHAIRE is a timed interval coding tool designed to code behaviors from video data. In this paper, we describe the development process of the OHAIRE, and present the results of analyses of its psychometric properties collected over four studies (10–12), including analyses of the OHAIRE's reliability, and validity.

Reliability refers to the property of a research tool to yield consistent results when used by different observers or at different times to assess the same situation. Good reliability indicators demonstrate that the tool provides enough details to parse out the subjectivity of the observer. The objectivity of an observer can be compromised by a number of sources of bias, such as the observer's familiarity with the individual whose behavior is being coded, either in the form of a personal relationship between the observer and the individual, or through the knowledge of some characteristics or demographics of an individual (e.g., socio-economic status, disease, or disorder diagnosis). Another source of observer bias can come from the knowledge of a study's design or hypotheses. In order to minimize the risk of bias, observers should be blinded to as many variables as possible that may influence their judgement, and given clear instructions on how to use the research tool. In this paper, we assess inter-rater reliability to test whether the OHAIRE manual contains precise and clear definitions and whether the training of coders was effective. Intra-rater reliability is assessed to measure the drift of coders' observations over time and the potential need for re-training (13).

Validity refers to the capacity of an instrument to generate data that is representative of the actual behaviors it intends to measure. Validity can be assessed using many different types of evidence. In this paper, we assess convergent and divergent validity of the OHAIRE by evaluating its correlation with standardized questionnaires. We expect that subscales of the OHAIRE will correlate with measures that assess similar constructs. We also explored the internal reliability of the subscales of the OHAIRE, or how coded behaviors from the same subscale relate to each other.

### Development of the OHAIRE coding system

In an effort to quantify human behaviors theorized to be generated by interacting with animals, the OHAIRE coding system was developed.

#### Behavior definitions

The choice of the behaviors to include in the OHAIRE was made based on a review of common behaviorally relevant variables reported in the HAI literature. The behaviors included can be observed in any naturalistic setting, whether the investigator is observing interactions between humans and animals in the home or during animal-assisted activities or therapy. In order to encompass common research questions in HAI research, the OHAIRE captures social interactions, interactions with animals, interactions with control objects, facial and verbal emotional display, and interfering behaviors. The list of behaviors is presented in Table 1.

**Table 1:** List of behaviors included in different versions of the OHAIRE.

**OHAIRE-V1**	**OHAIRE-V2**	**OHAIRE-V3**
**EMOTIONAL DISPLAY**		
**Facial Emotional Display**		
Smile	Smile	Smile
Laugh	Laugh	Laugh
Negative (Frown, Cry, Whine, Pain)	Negative (Frown, Cry)	Negative
	Neutral	None
**Verbal Emotional Display**		
Positive		Positive
Negative		Negative
None		None
**INTERACTIVE BEHAVIORS**		
**Social Communication**		
	Initiation vs. Response	
Talk (Peer, Adult, Unknown)	Talk (Peer, Adult)	Talk (Peer, Adult)
	Gesture (Peer, Adult)	Gesture (Peer, Adult)
Look (Peer, Adult)	Look (Peer, Adult)	Look (Peer, Adult)
Touch (Peer, Adult)	Touch (Peer, Adult)	Touch (Peer, Adult)
Affection (Peer, Adult)	Affection (Peer, Adult)	Affection (Peer, Adult)
Prosocial (Person)	Prosocial (Peer, Adult)	Prosocial (Peer, Adult)
**Interactions with Objects/Animals**		
Talk (Animal, Toy)	Talk (Animal, Object)	Talk (Animal, Object)
	Gesture (Animal, Object)	Gesture (Animal, Object)
Look (Animal, Toy)	Look (Animal, Object)	Look (Animal, Object)
Touch (Animal, Toy)	Touch (Animal, Object)	Touch (Animal, Object)
Affection (Animal, Toy)	Affection (Animal, Object)	Affection (Animal, Object)
Prosocial (Animal, Toy)	Prosocial (Animal, Object)	Prosocial (Animal, Object)
**PROBLEM BEHAVIORS**		
Aggression, Stealing	Aggression (Peer, Adult, Animal, Object, Self)	Aggression (Peer, Adult, Animal, Object, Self)
Disruption	Hyperactivity	Overactivity
Self-focused behavior, Leaving	Isolation, Anxiety, Sensory	Isolation
Other Problem Behaviors		

*V1, Version 1; V2, Version 2; V3, Version 3*.

Social interactions are a common outcome of interest in HAI research, from studies that evaluate the effect of being accompanied by a companion animal on social interactions with strangers [e.g., (14)], to the effect of animal-assisted intervention on the social skills of children with autism spectrum disorder [e.g., (15)]. The OHAIRE captures six different forms of social interactions, namely talking, looking, gesturing, touching, showing affection, and being prosocial (i.e., purposefully helpful) to others. The OHAIRE identifies the target of social interactions, whether they are directed toward adults or individuals of the same age cohort (i.e., peers) of research participants.

To account for interactions with animals, the OHAIRE captures the same behaviors toward animals. Following a push for more rigorous and controlled research in the field of HAI, more study designs have started to include active or attention control conditions to parse out the effect of the animal in a study. In an active or attention control condition, the participants engage in activities that mimic the amount of time and attention dedicated to participants in the treatment group. As these control conditions often include control objects, such as toys or stuffed animals, the OHAIRE captures the behaviors expressed toward these control objects.

Interacting with animals is also often reported to have a positive effect on mood and emotions [e.g., (16–18)]. To quantify this effect, the OHAIRE captures emotional display in two ways: facial emotional display and verbal emotional display. Facial emotional display refers to facial expressions of happiness, like smiling and laughing, and discontent or sadness, like frowning or crying. Verbal emotional display can be positive or negative, and refers to the valence of the speech of the participants; its coding relies on the actual words pronounced by the participant rather than on the tone of their voice.

Interfering behaviors coded with the OHAIRE encompass behaviors that may impair the individual's ability to participate in and benefit from an activity or interaction, including aggression, overactivity, and isolation. Aggression refers to any potentially harmful behaviors, and is coded along with its target (i.e., to whom or what it is directed). Overactivity is coded when a participant is loud, disruptive, or shows signs of restlessness. Isolation is coded when a participant is socially withdrawn, not engaged in their social environment.

All behaviors captured with the OHAIRE are described extensively in the OHAIRE coding manual. For each behavior, detailed coding tips and multiple examples are provided.

#### OHAIRE versions

Between its first use in 2013 (11), and the current paper, the OHAIRE has undergone modifications to improve the usability and psychometric properties of the tool. In total, three different versions of the OHAIRE were used over four studies, coded in six coding periods. Between the OHAIRE-Version 1 (OHAIRE-V1) and the OHAIRE-Version 2 (OHAIRE-V2), definitions of negative emotional display were simplified, gestures were added as a social communication behavior, interfering behaviors were simplified, and anxiety was added to the list of interfering behaviors. Between the OHAIRE-V2 and the OHAIRE-Version 3 (OHAIRE-V3), the definition of negative facial emotional display was further simplified, verbal emotional display was re-introduced, and interfering behaviors were re-arranged. The list of behaviors that were recorded for each version of the OHAIRE is available in Table 1. The mean, standard deviation, and skew of all behaviors are presented in Table 4. Overall, between the OHAIRE-v1 and the OHAIRE-v3, behaviors were added, removed, or merged in the tool, but the definitions of the behaviors were stable over time, which allows us to use data from all four studies coded with the OHAIRE so far for reliability and validity analyses.

## Methods

### Studies

The OHAIRE was used to assess the behavior of children in four independent HAI studies exploring the effects of animal-assisted intervention. A summary of the main characteristics of each study included in the analyses is presented in Table 2. The total combined sample for this paper included 201 children aged 5 to 18 (*M* = 10.1, *SD* = 2.5) and 2,732 min of coded video data.

**Table 2:** Summary of the studies included in the validity and reliability analyses.

	**Study 1**	**Study 2**	**Study 3**	**Study 4**
Site	The University of Queensland	University of Colorado, Denver	University of California, Irvine	Children's Hospital Colorado
Setting	Inclusion school classrooms	Riding facility	Developmental school	Psychiatric Hospital Unit
Species	Guinea pig	Horse	Dog	Dog
Sample size	33 + 66	19	36	47
Diagnosis	ASD & TD	ASD	ADHD	ASD
Age (years)	5 - 13	6 - 15	7 - 9	6 - 18
Treatment	AAA with Guinea pigs	Therapeutic Horseback Riding	CBT with dogs	AAA with dogs
Control	Play with toys	Barn activities	CBT with stuffed dogs	Play with toys
Coding	OHAIRE v1 & v2	OHAIRE v2	OHAIRE v3	OHAIRE v3

*ASD, Autism Spectrum Disorder; TD, Typically-developing; ADHD, Attention-Deficit Hyperactivity Disorder; AAA, Animal-Assisted Activities; CBT, Cognitive-Behavioral Therapy; OHAIRE, Observation of Human-Animal Interaction for Research*.

#### Study 1—species: guinea pigs, population: children with ASD and typically developing children

Study 1 assessed the effects of Animal-Assisted Activities (AAA) with guinea pigs in inclusion classrooms (11). Inclusion classrooms accommodate typically developing (TD) children as well as their peers with Autism Spectrum Disorder (ASD). Participants were recruited from 15 inclusion classrooms within four schools in the area of Brisbane, Australia. Thirty-three groups of three children participated in this program, each pairing one child with ASD with two TD children randomly selected from the same classroom (*N* = 99). Participants were aged 5 to 12 years old (*M* = 9.1, *SD* = 2.3) All groups participated in free-play sessions with toys and AAA sessions with guinea pigs. There were three 10-min free-play sessions with toys: one before an 8-week waitlist control, one after the waitlist and before an 8-week AAA program, and one at the end of the AAA program. The AAA program consisted of bi-weekly 20-min free-interaction sessions with guinea pigs and animal-related materials for 8 consecutive weeks. All sessions were video-recorded, and three toy sessions and three AAA sessions were selected for behavior coding. The first 10 min of each session was selected for coding. Results of this study indicated that children with ASD displayed more social behaviors, more positive affect, and less negative affect in the presence of animals, compared to toys (11). For TD children, results indicated more social behaviors, especially toward adults, and more positive emotional display in the presence of animals, compared to toys (19).

#### Study 2—species: horses, population: children with ASD

Study 2 assessed the effects of a Therapeutic Horseback Riding (THR) program for children with ASD (10). Sixteen participants ages 6 to 16 years (*M* = 10.2, *SD* = 3.0) were randomly assigned to a 10-week THR program or a 10-week control program of barn activities. Both conditions offered 45-min, once weekly sessions in small groups (2–4 participants). During the THR group, participants (*n* = 8) learned horsemanship and riding skills while engaged with a horse. The barn activity group participants (*n* = 8) learned similar horsemanship skills, but without contact with horses, instead activities involved a life size stuffed horse. Participants in this study were filmed for a minimum of 1 min before and after each intervention group (THR and barn activity), and all sessions were included in behavior coding. Participants in THR group were recorded before the group while waiting to ride seated on a bench on the side of the riding arena. Barn activity group participants were recorded while waiting for the group to begin while seated at the group table. Both group participants were recorded in similar conditions after the groups (i.e., seated at a table with their respective groups engaging with art materials). Because of the timing of the recordings, participants were not taped when interacting with horses or stuffed horses, thus the results for this study do not include interactions with animals and control objects, but do include all other behaviors normally coded with the OHAIRE (emotional display, social interactions, and interfering behaviors).

#### Study 3—species: dogs, population: children with ADHD

Study 3 evaluated the effect of the inclusion of a dog in a cognitive behavioral therapy (CBT) group program for children with Attention-Deficit Hyperactivity Disorder (ADHD; 12). Thirty-six children ages 7–9 years old (*M* = 7.9, *SD* = 0.72) with a diagnosis of ADHD were randomized to groups of six participants to either receive CBT in the presence of dogs (*n* = 18) or with stuffed, plush dogs (*n* = 18). Participants attended twice-weekly sessions (a total of 4 $\frac{1}{2}$ h per week) for 12 weeks, over 23 sessions. All sessions were video-recorded. Five sessions were selected for behavior coding (sessions 1, 7, 12, 18, and 23), with an attempt to maximize the number of participants present at each of the coded session, and to represent different sessions at regular intervals during the length of the intervention.

#### Study 4—species: dogs, population: children with ASD

Study 4 assessed the effect of a dog's presence on the behavior of youth with ASD and co-existing psychiatric diagnoses admitted to a developmental disability specialty psychiatric unit. A total of 76 children and adolescents with ASD aged 6 to 18 (*M* = 12.4, *SD* = 3.5) participated in this crossover design 10-min sessions of unstructured activities with either a dog and adult handler or a marble track toy and adult handler. Forty-seven children participated in both types of sessions, 23 children participated in sessions with the dog only, and six children with the marble track toy only. Children participated in the activities in groups of two or three, and an adult supervisor. All sessions were video-recorded and used for behavior coding.

### Ethical considerations

Written informed parental consent and oral child participant assent were obtained for all participants in the studies used in the present article. The protocols for video transfers between institutions and coding of the videos at the first author's institution were reviewed and approved by the Purdue Institutional Review Board (Approval #1410015340). Study 1 human-related protocols were reviewed and accepted by the University of Queensland's Human Ethics Committee (Approval # 2010001284) and animal-related protocols were reviewed and accepted by the University of Queensland's Animal Ethics Committee (Approval # SPH/057/11). Study 2 and 4 human-related protocols were reviewed and accepted by the Colorado Multiple Institutional Review Board (Study 2, Approval # 07-1148; Study 4, Approval # 15-1227) and animal-related protocols were considered exempt of review by the University of Colorado IACUC as no research was directly performed on the animals. Study 3 human-related protocols were reviewed and approved by the University of California Irvine Institutional Review Board (Approval # 2010-7679) and animal-related protocols were considered exempt of review by the University of California Irvine Institutional Animal Care and Use Committee as no research was directly performed on the animals.

### Behavior coding

#### Sampling method

**Figure 1:**
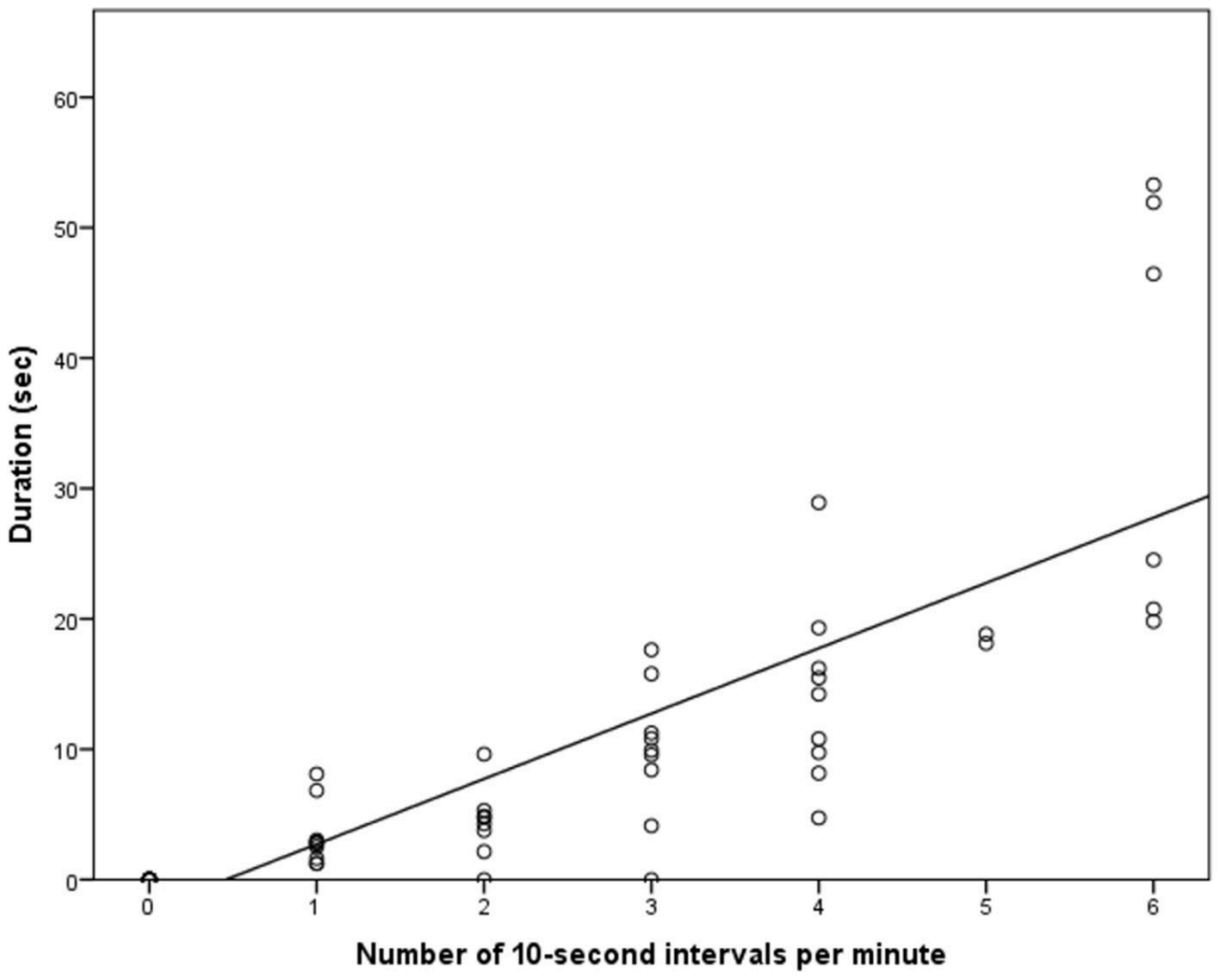
Relationship between one-zero sampling and the duration of time spent smiling while in the presence of toys or animals.

The OHAIRE coding system uses the online data entry system *Qualtrics* (20) to facilitate coding and reduce data entry error. The OHAIRE relies on the coding of 1-min video segments that are divided into six 10-s intervals. For each 10-s interval, behaviors are described as either present (1) or absent (0). The scores for each interval are summed to create a score out of six for a full minute for each behavior. This type of coding, called one-zero sampling or interval sampling, is an effective way to code large amounts of video data with high inter-rater reliability (21). In one-zero sampling, the behaviors are not *rated* in intensity, but rather *coded* as present or absent, thus, this technique is referred to as behavior coding, and the observers as coders. The lack of intensity rating and the coding as present or absent rather than an exact duration measurement are often cited as drawbacks of one-zero sampling; whereas its simple use yielding high reliability, and efficiency are cited as its major strengths [e.g., (22, 23)]. To verify the accuracy of one-zero sampling in our sample, we compared its use with measuring the exact duration of behaviors. To reduce time burden, we selected one behavior for one coder to measure using both one-zero sampling, and exact duration measurement in a randomly selected set of 60 one-min videos. We selected the behavior “smiling,” because it is common, but varies largely between children and videos. We selected videos from study 1 to compare one-zero sampling and duration measurement because this study had excellent video quality. Study 1 also included both ASD and TD children, which increased variability. A coder viewed 60 videos of children (30 ASD; 30 TD) from Study 1. Using a Spearman rank correlation to accommodate the ordinal one-zero sampling data, we found an excellent correlation (*r* = 0.92, *p* < 0.001) between the two sampling techniques (Figure 1). Additionally, the coder went through one-zero sampling faster than duration measurement, and reported feeling more confident with the judging criteria for one-zero sampling than for duration measurement. We concluded that with high reliability, high efficiency, and little loss in information, one-zero sampling is suited for use with the OHAIRE to address the current state of research in HAI, as proof-of-concept is still needed for numerous research questions.

#### Training

Each new coder undergoes a standardized training to learn to use the OHAIRE coding system. The training starts with a detailed study of the manual and the viewing of example videos for each behavior. Coders are then taught how to use the online coding system and the video sampling procedure. Next, coders are trained to code with videos from the specific study they will be working on. Since HAI is a broad field with different populations and types of interactions, coders should reach inter-rater reliability on a sample of the specified study's data before starting to code. The trainer and the coders first code a full minute of video together. Then, each coder views and then codes three videos by him or herself. After coding three videos, inter-rater reliability with the trainer is calculated. Differences in coding are discussed, and three more videos are coded. Cycles of coding three videos and subsequently discussing reliability continue until each coder has reached excellent overall inter-rater reliability (Cohen's Kappa > 0.8). This initial phase of training typically takes 3 to 5 h. Training will be made available to a larger public in the Spring of 2019. For more information, please visit http://www.ohairecoding.com.

#### Coders

For each study, one primary coder was designated to code the full set of videos. The data obtained from the primary coder was used for the scoring of the OHAIRE and the outcome data analyses. Additionally, one or more secondary coders coded at least 20% of the videos to calculate inter-rater reliability. Videos coded for reliability were selected randomly from the main coding sets with a random number generator. A total of 14 coders were trained in and used the tool. Coders are individually referred to as the letter “C” followed by a number between 1 and 14 for the rest of this article.

### Questionnaires

Each study included standardized informant-report questionnaires. We decided to focus on questionnaires that had been used in at least two studies to explore the convergent and divergent validity of the OHAIRE. Questionnaires included in each study are listed in Table 3.

**Table 3:** Questionnaires included from each of the studies.

	**Study 1**	**Study 2**	**Study 3**	**Study 4**
ABC		X		X
SCQ	X	X		
SRS		X		X
SSRS	X		X	

*ABC, Aberrant Behavior Checklist; SCQ, Social Communication Questionnaire; SRS, Social Responsiveness Scale; SSRS, Social Skills Rating System*.

#### Aberrant behavior checklist

The Aberrant Behavior Checklist-Community [ABC-C; (24)], is a 58-item questionnaire developed to assess interfering behaviors in children and adults with intellectual and developmental disabilities. The ABC comprises five subscales, including irritability and agitation, lethargy and social withdrawal, stereotypic behavior, hyperactivity and non-compliance, and inappropriate speech. In multiple studies, the ABC-C has shown high internal consistency, good inter-rater reliability, and a consistent five-factor structure [e.g., (25, 26)]. Higher ABC-C scores indicate more aberrant behaviors.

The ABC-C was used in Study 2 and Study 4 for two purposes: As a screening measure for entry in the studies, and as a weekly outcome measure using the irritability subscale (10). For consistency, we used only the first ABC-C score recorded for each child (baseline score) in the present analyses. In both studies, the ABC-C was completed by a caregiver for each child.

#### Social communication questionnaire

The Social Communication Questionnaire [SCQ; (27)], is a 40-item questionnaire developed to assess autism-like behavior in individuals of all chronological ages and with a developmental age over 2 years. The SCQ demonstrates good internal consistency, test-retest reliability, and convergent reliability with other ASD diagnostic tools (27). Higher SCQ scores indicate more behaviors characteristic of ASD.

The SCQ-Lifetime was completed in both Study 1 and Study 2 by caregivers of the participants upon entry in the study, as an additional screening measure for ASD.

#### Social responsiveness scale

The Social Responsiveness Scale [SRS; (28)] is a 65-item rating scale developed to measure symptoms associated with autism spectrum disorder. The SRS comprises five subscales, namely social awareness (eight items), social cognition (12 items), social communication (22 items), and social motivation (11 items), which can be summarized in an overall Social subscale score, and Restricted and Repetitive Behaviors. The SRS demonstrates high internal consistency and test-retest reliability (29). Its updated version, the SRS-2, enlarges the age range of the intended SRS test-taking population (30). Higher SRS scores indicate more problems in the designated subscale.

Participants' caregivers completed the SRS in Study 2, and the SRS-2 in Study 4. For the age ranges of participants included in this paper the SRS-2 does not introduce new subscales or items, therefore scores of the SRS and SRS-2 will be presented together in the subsequent analyses. In both studies, questionnaires were completed upon entry in the study and after the intervention period. For consistency, we used the SRS and SRS-2 scores of participants at study entry for the validity analyses in this paper.

#### Social skills rating system

The Social Skills Rating System [SSRS; (31)] is a 57-item (teacher version) or 55-item (parent version) rating scale developed to measure Social Skills and Competing Problem Behaviors as rated by parents or teachers, and academic competence as rated by teachers in children. The SSRS demonstrates adequate internal consistency and test-retest reliability (31). Its updated version, the Social Skills Improvement System [SISS; (32)], is a 79-item measure structured similarly, with additional subscales and improved psychometric properties. Because scores on the social skills and problem behavior scales of the SSRS and the SSIS are highly correlated (33), these scores will be presented together in the subsequent analyses.

In Study 1, the SSRS was completed by parents and teachers of participants upon entry in the study, after an 8-week waitlist period, and after an 8-week program of animal-assisted activities. In Study 3, the SSIS was completed by parents of participants upon entry in the study, at the end of the intervention period, and at a 6-week follow-up. For consistency, SSRS and SSIS scores from the time of study entry are used for validity analyses in this paper. Higher scores indicate better skills in the social skills and academic competence subscales of the SSRS and SSIS, while higher scores indicate more problem behaviors in the competing problem behavior subscale.

### Data analyses

#### Inter-rater reliability

Ensuring that the observation coding tool was used consistently across coders was important to parse out coders' subjectivity, which may reflect the quality of the training and the precision of the manual. To assess inter-rater agreement, a primary coder coded all (100%) of the videos for each study, and one or two secondary coders coded 20% of the videos or more. We calculated Cohen's kappa (34), an agreement coefficient that corrects for chance agreement. Cohen's kappa values range from −1, indicating complete disagreement, to 1, indicating perfect agreement. In this paper, we base our interpretation of kappa values on recent guidelines, considering values above 0.20 minimal, above 0.40 weak, above 0.60 moderate, above 0.80 strong, and above 0.90 excellent (35).

#### Intra-rater reliability

Observer drift can be an issue observed in the days or week following initial inter-rater reliability training, which can result in observers coding behaviors with less accuracy (13, 36). To assess the risk of observer drift in the OHAIRE, we calculated intra-rater reliability for a random selection of videos from all four studies included in this paper. Coders were assigned a list of 30 videos to code in 1 week, then again 2 weeks later. We calculated Cohen's kappa between the two coding repetitions for each study. We used McHugh's interpretation of Cohen's kappa for intra-rater reliability (35).

#### Convergent and divergent validity

We examined potential correlations of the OHAIRE with questionnaire data to provide evidence of convergent and divergent validity. We compared the average OHAIRE score of each participant with the ABC-C, the SCQ, the SRS and SRS-2, and the SSRS and SSIS scores upon entry in studies. For all questionnaires, raw scale and subscale scores were used. OHAIRE behavior scores of facial emotional display, verbal emotional display, and interfering behaviors were included individually in the analyses. OHAIRE scores of social interactions with peers, social interactions with adults, interactions with animals (human-animal bond score), and interactions with objects were included as subscale scores. Pearson's correlations were used to adapt to the continuous rating scales of the questionnaires, and mean OHAIRE values per participant ranging in a near-continuous way from 0 to 6. We hypothesized the following correlations:

1. [1.] Aberrant Behavior Checklist – Community
  - [(1)] Irritability and Agitation subscale correlated negatively with positive facial and verbal emotional display, and positively with negative facial and verbal emotional display.
  - [(2)] Lethargy and Social Withdrawal subscale correlated negatively with social interactions with peers and adults, and positively with social isolation.
  - [(3)] Stereotypy and Hyperactivity subscales correlated positively with overactivity.
  - [(4)] Inappropriate speech subscale correlated positively with aggression.
1. [2.] Social Communication Questionnaire
  - [(1)] SCQ scores correlated negatively with positive facial expressions (smile, laugh), and social interactions with peers and adults.
  - [(2)] SCQ scores correlated positively with negative facial expressions and overactivity.
1. [3.] Social Responsiveness scale
  - [(1)] Social subscale correlated negatively with OHAIRE scores of social interactions with peers and adults, and positively with isolation.
  - [(2)] Restricted interests and repetitive behaviors subscale correlated positively with overactivity.
1. [4.] Social Skills Rating System
  - [(1)] Social skills scale correlated positively with OHAIRE scores of social interactions with peers and adults, and negatively with isolation.
  - [(2)] Competing problem behaviors scale correlated negatively with OHAIRE scores of social interactions with peers and adults, and positively with OHAIRE scores of aggression, overactivity, and isolation.

#### Structure

The behaviors coded in the OHAIRE were originally arranged in behavioral categories designed to facilitate ease of coding (i.e., emotional display, interactive behaviors, and interfering behaviors), rather than designed to be used as aggregate subscales. While the behavioral categories “emotional display” and “interfering behaviors” consist of unique behaviors that have distinct functions, behaviors coded in the category “interactive behaviors” refer to the common function of interacting with either a peer, an adult, an animal, or an object. We used Cronbach's alpha (37) to assess the internal consistency of the following subscales for the OHAIRE: social interactions with adults, social interactions with peers, interactions with animals, and interactions with objects. We used average OHAIRE scores for each participant.

## Results

The descriptive statistics for behavioral codes of the OHAIRE across all studies, averaged by child and then by study, are presented in Table 4.

**Table 4:** Descriptive statistics of OHAIRE behavioral codes, averaged by individual.

**OHAIRE behavioral code**	**N**	**Observed range**	**Mean**	**(SD)**	**Skew**
**FACIAL EMOTIONAL DISPLAY**					
Smile	213	0–6	1.89	(1.21)	0.73
Laugh	213	0–4.72	0.37	(0.55)	3.70
Negative	213	0–4	0.12	(0.36)	7.11
None	180	0–6	4.48	(1.3)	−0.95
**VERBAL EMOTIONAL DISPLAY**					
Positive	129	0–2.33	0.34	(0.45)	1.75
Negative	129	0–0.61	0.05	(0.12)	2.67
None	129	0–6	4.32	(2.44)	−1.05
**SOCIAL INTERACTIONS**					
With peers	213	0.17–6	2.63	(1.28)	0.17
Talk	213	0–4.11	0.98	(0.88)	0.98
Gesture	180	0–1.22	0.25	(0.28)	1.25
Look	213	0–4.89	1.82	(1.21)	0.43
Touch	213	0–5	0.82	(0.8)	1.46
Affection	213	0–0.14	0.00	(0.01)	8.43
Prosocial	180	0–1.22	0.09	(0.19)	3.24
With adults	213	0–5.67	2.37	(1.21)	0.34
Talk	213	0–4.5	1.32	(0.99)	0.97
Gesture	180	0–2.57	0.40	(0.41)	1.94
Look	213	0–5.5	1.60	(1.05)	1.04
Touch	213	0–2.06	0.31	(0.37)	1.98
Affection	213	0–0.87	0.01	(0.07)	8.70
Prosocial	180	0–1.14	0.08	(0.2)	3.08
**INTERACTIONS WITH ANIMALS OR OBJECTS**					
With animals	213	0–6	1.98	(1.38)	0.29
Talk	213	0–3.33	0.12	(0.36)	5.51
Gesture	180	0–1.33	0.04	(0.14)	5.96
Look	213	0–6	1.90	(1.22)	0.58
Touch	213	0–5.67	1.55	(1.1)	0.59
Affection	213	0–5.67	1.05	(1.03)	1.20
Prosocial	213	0–3	0.81	(0.99)	0.77
With objects	213	0–6	3.34	(1.96)	−0.39
Talk	180	0–1.17	0.02	(0.1)	9.09
Gesture	180	0–0.67	0.01	(0.06)	8.05
Look	213	0–6	3.06	(2.13)	−0.30
Touch	213	0–6	2.47	(1.71)	−0.04
Affection	213	0–1.17	0.04	(0.16)	4.48
Prosocial	180	0–0.06	0.00	(0)	13.42
**PROBLEM BEHAVIORS**					
Aggression	213	0–1.17	0.03	(0.11)	7.05
To peer	213	0–0.5	0.01	(0.04)	8.74
To adult	213	0–0.06	0.00	(0.01)	10.30
To animal	213	0–0.22	0.00	(0.02)	14.59
To object	213	0–1.17	0.02	(0.09)	9.77
To self	180	0–0.17	0.00	(0.02)	9.41
Overactivity	213	0–4.83	0.49	(0.92)	2.18
Isolation	213	0–4.44	0.50	(0.78)	2.09

### Inter-rater reliability

The number of videos coded by primary and secondary coders for each study, as well as overall Cohen's kappa between pairs of coders for the OHAIRE coding system and for five categories of behaviors are presented in Table 5. Overall, inter-rater reliability was excellent (0.79 < k < 0.88), with differences across behavior categories. Facial and verbal emotional display are coded with moderate to excellent agreement (0.62 < k < 0.99), and interfering behaviors yield strong to excellent agreement across all studies (0.88 < k < 0.98). Social communication yields weak to moderate agreement in most studies (0.37 < k < 0.79), with a drop in kappa for the TD sample of Study 1. Interactions with animals and objects yields moderate to excellent reliability in most studies (0.67 < k < 0.91), except for Study 2 (*k* = 0.16). The most recent version of the coding system (OHAIRE-V3), used in Studies 3 and 4, yielded moderate to excellent inter-rater reliability in all categories.

**Table 5:** Inter-rater reliability results.

	**Study 1**			**Study 2**		**Study 3**	**Study 4**
	**ASD subsample**	**TD subsample**		***N =* 3 subsample**	***N =* 16 subsample**		
**OHAIRE Version**	**v1**	**v2**	**v2**	**v2**	**v2**	**v3**	**v3**
**Coder ID**	**C1 & C2**	**C3 & C4**	**C3 & C5**	**C6 & C7**	**C8 & C9**	**C10 & C11**	**C12 & C13**
**SAMPLE**							
Number of videos coded by the primary coder	594	1174	1174	64	232	328	340
Number of videos coded by the secondary coder	238	87	162	15	54	139	78
Percent of videos coded for reliability	40%	7.4%	13.8%	23,4%	23.3%	42.4%	22.9%
**RELIABILITY**							
Overall	**0.79**	**0.82**	**0.81**	**0.88**	**0.81**	**0.83**	**0.83**
Facial Emotional Display	**0.74**	**0.71**	**0.69**	**0.66**	**0.80**	**0.67**	**0.71**
Verbal Emotional Display	**0.62**	–	–	–	–	**0.99**	**0.86**
Social Communication	**0.73**	0.37	**0.41**	**0.79**	**0.65**	**0.56**	**0.68**
Interactions with animals and objects	**0.91**	**0.82**	**0.67**	**0.73**	0.16	**0.73**	**0.85**
Problem Behaviors	**0.90**	**0.98**	**0.98**	**0.96**	**0.90**	**0.88**	**0.95**

*ASD, Autism Spectrum Disorder; TD, Typically-developing; all ps < 0.001. Bold face font indicates acceptable inter-rater reliability (k > 0.40)*.

### Intra-rater reliability

Intra-rater reliability for coding occasions separated by 2 weeks was calculated for a subset of 26 to 30 videos by study. Overall intra-rater reliability was excellent, with Cohen's kappa varying between 0.87 and 0.96 (Table 6). Intra-rater reliability was moderate to excellent across five behavior categories, with slightly lower reliability for social communication (0.72 < k < 0.88), and excellent agreement for interfering behaviors (0.97 < k < 0.98). Intra-rater reliability seems to vary between coders, with notably one coder who performed slightly worse than others, with a strong kappa of 0.87, compared to excellent kappas (above 0.90) for all other three coders (C13, Study 4).

**Table 6:** Cohen's Kappa values for intra-rater reliability results.

	**Study 1**	**Study 2**	**Study 3**	**Study 4**
**Coder ID**	**C14**	**C10**	**C10**	**C13**
Number of videos	26	28	30	30
Overall	0.90	0.95	0.96	0.87
Facial Emotional Display	0.83	0.86	0.91	0.77
Verbal Emotional Display	0.99	0.97	0.99	0.83
Social Communication	0.74	0.86	0.88	0.72
Interactions with animals and objects	0.81	0.86	0.82	0.74
Problem Behaviors	0.98	0.97	0.97	0.98

### Convergent and divergent validity

#### Aberrant behavior checklist—community

Pearson's correlations between the OHAIRE behavior scores and the ABC-C scores are summarized in Table 7. Contrarily to our hypotheses, the Irritability and Agitation subscale did not correlate significantly with positive facial and verbal emotional display. It correlated positively with negative facial display for Study 2 but not for Study 4. Contrarily to our hypotheses, the Lethargy and Social Withdrawal subscale was correlated negatively with social interactions with adults only in Study 4, and was not correlated positively with social isolation. Additionally, the ABC-C Lethargy Social Withdrawal subscales were negatively correlated with interactions with animals and over activity for Study 2. Contrarily to our hypotheses, the Stereotypy and Hyperactivity subscales were not correlated positively with the OHAIRE over activity scale, but ABC-C Hyperactivity was correlated positively with negative emotional display (*r* = 0.72, *p* < 0.001) and interactions with adults (*r* = 0.67, *p* = 0.003), and negatively with social isolation (*r* = −0.52, *p* = 0.033). Contrarily to our hypotheses, the Inappropriate Speech subscale did not correlate positively with aggression.

**Table 7:** Pearson's correlations between the OHAIRE coding system and the Aberrant Behavior Checklist-Community Subscales.

**ABC-C subscales**	**Irritability, Agitation**		**Lethargy, Social withdrawal**		**Stereotypy**		**Hyperactivity**		**Inappropriate speech**	
**Study**	**S2**	**S4**	**S2**	**S4**	**S2**	**S4**	**S2**	**S4**	**S2**	**S4**
**N**	**17**	**49**	**17**	**49**	**17**	**49**	**17**	**49**	**17**	**49**
**FACIAL EMOTIONAL DISPLAY**										
Smile	0.00	−0.06	−0.08	−0.18	0.04	−0.03	−0.05	−0.08	0.28	0.08
Laugh	0.21	−0.07	0.24	−0.08	−0.13	0.10	0.44^†^	−0.01	0.16	−0.06
Negative	0.57^*^	0.03	0.45^†^	−0.08	−0.01	−0.14	0.72^***^	0.15	0.29	0.25^†^
None	−0.21	0.04	−0.15	0.22	−0.04	0.11	−0.24	0.01	−0.44^†^	−0.17
**VERBAL EMOTIONAL DISPLAY**										
Positive	–	−0.01	–	−0.08	–	−0.07	–	0.06	–	0.01
Negative	–	−0.15	–	0.17	–	−0.01	–	0.06	–	−0.04
None	–	0.04	–	0.04	–	0.07	–	−0.04	–	0.01
**SOCIAL INTERACTIONS**										
With Peers	0.32	0.01	0.02	−0.03	−0.08	−0.18	0.25	−0.01	0.13	−0.23
With Adults	0.45^†^	−0.24^†^	−0.19	−0.31^*^	0.17	0.05	0.67^**^	0.01	0.44^†^	−0.05
**INTERACTIONS WITH ANIMALS OR OBJECTS**										
With Animals	−0.03	−0.15	−0.51^*^	−0.13	0.00	−0.07	0.17	0.00	−0.12	−0.02
With Objects	0.38	−0.02	−0.18	0.19	0.20	0.05	0.48^†^	0.01	0.41	−0.21
**PROBLEM BEHAVIORS**										
Aggression	0.06	−0.06	−0.04	−0.10	0.10	−0.12	−0.11	−0.02	0.12	0.06
Overactivity	0.09	−0.07	−0.50^*^	−0.15	0.38	−0.06	−0.02	−0.02	0.13	0.00
Isolation	−0.35	−0.04	0.23	−0.11	0.03	−0.08	−0.52^*^	−0.03	−0.32	0.12

† *p < 0.10*;

* p < 0.05;

** p < 0.01;

*** *p < 0.001*.

#### Social communication questionnaire

Pearson's correlations between the OHAIRE behavior scores and the SCQ scores are summarized in Table 8. Confirming our hypothesis, SCQ scores did significantly correlate negatively with positive facial expressions (smile, *r* = −0.56, *p* < 0.001; laugh, *r* = −0.21, *p* = 0.049), and with social interactions with peers (*r* = −0.50, *p* < 0.001), although not with adults for Study 1. SCQ scores correlated positively with negative facial expressions as hypothesized (*r* = 0.34, *p* = 0.001), but contrarily to our hypothesis, not with overactivity for Study 1. Trends were overall the same for Study 2, without reaching statistical significance. Additionally, the SCQ correlated positively with interactions with animals (*r* = 0.43, *p* < 0.001) and objects (*r* = 0.42, *p* < 0.001), and aggression (*r* = 0.40, *p* < 0.001), and negatively with isolation (*r* = −0.52, *p* < 0.001) in Study 1. It correlated negatively with interactions with objects (*r* = −0.61, *p* < 0.001) in Study 2.

**Table 8:** Pearson's correlations between the OHAIRE coding system and the Social Communication Questionnaire.

	**SCQ**	
**Study**	**S1**	**S2**
**N**	**90**	**16**
**FACIAL EMOTIONAL DISPLAY**		
Smile	−0.56^***^	−0.49^†^
Laugh	−0.21^*^	0.12
Negative	0.34^**^	0.23
None	0.04	0.40
**VERBAL EMOTIONAL DISPLAY**		
Positive	−0.29	–
Negative	0.24	–
None	0.10	–
**SOCIAL INTERACTIONS**		
With Peers	−0.50^***^	−0.27
With Adults	0.12	−0.24
**INTERACTIONS WITH ANIMALS OR OBJECTS**		
With Animals	0.43^***^	−0.22
With Objects	0.42^***^	−0.61^*^
**PROBLEM BEHAVIORS**		
Aggression	0.40^***^	−0.16
Overactivity	0.18~	−0.13
Isolation	−0.52^***^	0.37

† p < 0.10;

* p < 0.05;

** p < 0.01;

*** *p < 0.001*.

#### Social responsiveness scale

Pearson's correlations between the OHAIRE behavior scores and the SRS scores are summarized in Table 9. Contrarily to our hypotheses, no statistically significant correlations were observed between the SRS and OHAIRE behavior scores. Overall tendencies show a possible positive association between the Restricted Interests and Repetitive Behaviors Subscale and negative facial emotional display, and a negative association with positive facial emotional display. The Social subscale did not correlate negatively with OHAIRE scores of social interactions with peers and adults, and positively with isolation, and the Restricted Interests and Repetitive Behaviors subscale did not correlate positively with over activity.

**Table 9:** Pearson's correlations between the OHAIRE coding system and the Social Responsiveness Scale.

	**Social Subscale**		**Restricted Interests and Repetitive Behaviors Subscale**	
**Study**	**S2**	**S4**	**S2**	**S4**
**N**	**18**	**40**	**17**	**40**
**FACIAL EMOTIONAL DISPLAY**				
Smile	0.06	0.20	−0.25	−0.03
Laugh	0.10	−0.06	0.17	−0.31^†^
Negative	0.08	−0.16	0.43^†^	0.18
None	−0.17	−0.05	−0.03	−0.17
**VERBAL EMOTIONAL DISPLAY**				
Positive	–	−0.08	–	−0.10
Negative	–	0.04	–	0.21
None	–	0.06	–	0.05
**SOCIAL INTERACTIONS**				
With Peers	−0.01	−0.26	0.04	−0.23
With Adults	−0.07	0.19	0.30	−0.05
**INTERACTIONS WITH ANIMALS OR OBJECTS**				
With Animals	−0.46^†^	−0.17	0.10	−0.22
With Objects	0.03	0.14	0.27	0.16
**PROBLEM BEHAVIORS**				
Aggression	0.01	−0.02	0.30	−0.18
Overactivity	0.01	−0.16	0.28	−0.22
Isolation	−0.08	0.12	−0.07	0.16

† p < 0.10;

^*^p < 0.05; ^**^p < 0.01; ^***^p < 0.001.

#### Social skills rating system

Pearson's correlations between the OHAIRE behavior scores and the SSRS and SSIS scores are summarized in Table 10. In Study 1, the Social Skills scale of the SSRS as rated by parents and teachers was positively correlated with OHAIRE scores of social interactions with peers as hypothesized (parent, *r* = 0.42, *p* < 0.001; teacher, *r* = 0.28, *p* = 0.006), but, contrarily to our hypothesis, it was not correlated with social interactions with adults, and it was positively correlated with isolation (parent, *r* = 0.39, *p* < 0.001; teacher, *r* = −0.44, *p* < 0.001). Additionally, the Social Skills scale of the SSRS was positively correlated with smiling (parent, *r* = 0.51, *p* < 0.001; teacher, *r* = 0.42, *p* < 0.001), negatively correlated with negative facial emotional display (parent, *r* = −0.23, *p* = 0.031; teacher, *r* = −0.30, *p* = 0.003) and negative verbal emotional display (parent, *r* = −0.19, *p* = 0.303; teacher, *r* = −0.35, *p* = 0.043), and negatively correlated with interactions with animals (parent, *r* = −0.33, *p* = 0.001; teacher, *r* = −0.30, *p* = 0.003) and objects (parent, *r* = −0.28, *p* = 0.006; teacher, *r* = −0.40, *p* < 0.001). In Study 3, the Social Skills scale of the SSIS was not correlated with emotional display or social interactions, but was positively correlated with OHAIRE behavior scores of aggression (*r* = 0.44, *p* < 0.001).

**Table 10:** Pearson's correlations between the OHAIRE coding system and the Social Skills Rating System and the Social Skills Improvement System.

	**Social Skills Scale**			**Competing Problem Behaviors**			**Academic Competence**
**Study**	**S1**		**S3**	**S1**		**S3**	**S1**
**Version**	**SSRS**		**SSIS**	**SSRS**		**SSIS**	**SSRS**
**Rater**	**Parent**	**Teacher**	**Parent**	**Parent**	**Teacher**	**Parent**	**Teacher**
**N**	**91**	**97**	**36**	**91**	**97**	**36**	**97**
**FACIAL EMOTIONAL DISPLAY**							
Smile	0.51^***^	0.42^***^	0.20	−0.41^***^	−0.41^***^	0.04	0.31^**^
Laugh	0.20^†^	0.15	−0.03	−0.10	−0.11	−0.08	0.16
Negative	−0.23^*^	−0.30^**^	0.18	0.14	0.27^**^	−0.09	−0.13
None	−0.06	−0.01	−0.26	−0.04	0.18	−0.01	0.19
**VERBAL EMOTIONAL DISPLAY**							
Positive	0.06	−0.01	0.15	0.09	0.03	−0.11	−0.05
Negative	−0.19	−0.35^*^	−0.22	−0.17	0.42^*^	0.15	−0.08
None	0.03	−0.34^†^	−0.07	0.10	0.28	0.06	−0.16
**SOCIAL INTERACTIONS**							
With Peers	0.42^***^	0.28^**^	0.07	−0.23^*^	−0.20^*^	−0.21	0.19^†^
With Adults	−0.14	−0.11	0.06	0.12	0.15	−0.15	−0.11
**INTERACTIONS WITH ANIMALS OR OBJECTS**							
With Animals	−0.33^**^	−0.30^**^	−0.27	0.24^*^	0.23^*^	0.06	−0.32^**^
With Objects	−0.28^**^	−0.40^***^	0.32^†^	0.36^***^	0.37^***^	−0.38^*^	−0.34^***^
**PROBLEM BEHAVIORS**							
Aggression	−0.32^**^	0.24	0.24^*^	−0.20	−0.44^***^	0.44^***^	−0.29^**^
Overactivity	−0.16	−0.19^†^	0.34^*^	0.08	0.07	−0.16	−0.16
Isolation	0.39^***^	0.44^***^	−0.16	−0.48^***^	−0.40^***^	0.01	0.38^***^

*SSRS, Social Skills Rating System; SSIS, Social Skills Improvement System*.

† p < 0.10;

* p < 0.05;

** p < 0.01;

*** *p < 0.001*.

In Study 1, the Competing Problem Behaviors scale of the SSRS was correlated negatively with OHAIRE scores of social interactions with peers as hypothesized (parent, *r* = −0.23, *p* = 0.031; teacher, *r* = −0.20, *p* = 0.045), but unexpectedly not with adults. It was also, contrarily to our hypotheses, not correlated with overactivity, and positively correlated with aggression (parent, *r* = 0.24, *p* = 0.024; teacher, *r* = −0.44, *p* < 0.001), and isolation (parent, *r* = −0.48, *p* < 0.001; teacher, *r* = −0.40, *p* < 0.001). Additionally, the Competing Problem Behaviors scale of the SSRS was positively correlated with interactions with animals (parent, *r* = 0.24, *p* = 0.020; teacher, *r* = 0.23, *p* = 0.026), and objects (parent, *r* = 0.36, *p* < 0.001; teacher, *r* = −0.37, *p* < 0.001). In Study 3, the Competing Problem Behaviors subscale was not correlated with OHAIRE behavior scores of facial emotional display or social interactions, but was negatively correlated with interactions with objects (*r* = −38, *p* = 0.021).

Finally, the Academic Competence subscale of the SSRS was positively correlated with OHAIRE behavior scores of smiling (*r* = 0.31, *p* = 0.002), and isolation (*r* = 0.38, *p* < 0.001), and negatively correlated with OHAIRE behavior scores of interactions with animals (*r* = −0.32, *p* = 0.001) and objects (*r* = −0.34, *p* = 0.001), and aggression (*r* = −0.29, *p* = 0.004).

### Structure

Cronbach's alphas were moderate for subscales of social interactions with peers (α = 0.638), social interactions with adults (α = 0.605), interactions with animals (α = 0.773), and low for interactions with objects (α = 0.589).

## Discussion

The OHAIRE coding tool was developed to fill a need for a standardized behavior observation method in the field of HAI. In this article, we presented analyses of its reliability and validity, and summarized changes to the tool implemented to improve its psychometric properties, in the OHAIRE-V2, and OHAIRE-V3.

Overall, the OHAIRE demonstrated good inter-rater reliability, with variability between behavioral categories and increasing reliability through the versions of the OHAIRE. Intra-rater reliability was excellent but varied slightly between coders. Correlational analyses showed limited concordance between the behaviors coded with the OHAIRE during animal-assisted intervention, and questionnaires measuring various aspect of social communication, interfering behaviors, and ASD symptoms. These correlations varied widely across studies and questionnaires. Analyses of subscale internal consistency showed predominantly low to moderate Cronbach's alpha values.

The inter-rater reliability of the OHAIRE was overall excellent but varied with the version of the tool used, and peaked in the latest version of the tool, the OHAIRE-v3. Low inter-rater reliability was interpreted as a lack of precision of the coding manual, and following inter-rater reliability analyses, changes were made to increase its clarity. For example, the notions of initiation and response of interactions that was included in the OHAIRE-v2 led to confusion, and apart from expert raters (RG & MG), it yielded low inter-rater reliability for social communication and interactions with objects or animals. Only the form of interaction (talk, gesture, etc.) was retained for analyses in the current paper, and for the next version of the tool. The latest version of the tool, the OHAIRE-v3, shows improved reliability from previous versions in all behavioral categories.

In addition to imprecisions in the earlier versions of the coding manual, one reason for lower inter-rater reliability may be the personal performance of coders. The calculation of intra-rater reliability indicated how well coders retain their training and whether some behavior definitions are more or less likely to drift over time. While all coders retained excellent reliability over time, one coder scored slightly lower than others in all categories (except for interfering behaviors), despite having received the same training. This difference highlights the need for precise recruitment and in-depth training.

Analyses of the convergence of the OHAIRE with standardized questionnaires showed varying correlations depending on the questionnaire and the sample tested. Overall, our hypotheses as to the direction of correlations between the OHAIRE and varying questionnaires were not validated. One important factor of variation in correlations was the study that was tested. For example, the SCQ and the SSRS show strong correlations with the OHAIRE as used in Study 1, but much less so for Study 2 (SCQ) and Study 3 (SSRS). This difference is likely due to the difference in samples between studies. While Study 1 had a mixed sample of TD children and children with ASD from inclusion classrooms, both Study 2 and Study 3 had samples of participants enrolled in a treatment program for one particular neurodevelopmental disorder (ASD and ADHD, respectively). Specifically, there are strong correlations between the “interactions with animals” subscale of the OHAIRE and the SCQ and SSRS in Study 1, which is consistent with differences in SCQ and SSRS scores between children with ASD and TD children in this sample, and more interactions with animals displayed by children with ASD compared to TD children in this study (11, 19). The lack of correlations between questionnaires and behaviors coded with the OHAIRE may reflect a lower variance in these populations. For example, a minimum SCQ score was required for children with ASD to be able to participate in Study 2. If all children have SCQ scores in a restricted range, it may be expected that we see weaker or no correlations with the OHAIRE.

Another important consideration is that the OHAIRE directly evaluates the behavior of children during interventions. The questionnaires used in correlation analyses were mostly completed by caregivers, asking retrospective questions about the recent behavior of their child. However, behavior can vary widely from one setting to the other (38), and we do expect it to vary when the child is participating in animal-assisted intervention sessions. In the future, the correlation of behaviors as coded with the OHAIRE and change scores in questionnaires for before to after an intervention might help to explain how a child particularly benefited from a given intervention. The comparison of behavioral data with continuous physiological data, such as electrodermal activity or heart rate variability, may also provide evidence of convergent validity of the OHAIRE with another direct measure.

In addition to observing child behavior, recording the behavior of an animal in animal-assisted intervention may provide a more complete picture of human-animal interaction, including animal welfare. The dyadic analyses of the behavior of a human study participant and an animal may help identify specific activities with the animal or behaviors of the animal that trigger certain responses in a child. The development of animal behavior modules for species often included in animal-assisted intervention (e.g., dogs, horses) is a next step in the development of the OHAIRE.

Analyses of internal consistency with Cronbach's alpha yielded preliminary support for the use of four interaction subscales: social interactions with peers, social interactions with adults, interactions with animals (i.e., human-animal bond score), and interacting with a toy or control object. Specifically, the subscale measuring interactions with animals shows high internal consistency and can be used to quantify the engagement of a study participant with animals. This behavioral human-animal bond score may also be used in the future as a potential moderator of animal-assisted intervention success. For example, future studies may use the behavioral human-animal bond score as a way to explore whereas an animal-assisted intervention's success depends on the actual level of engagement of its participants with animals, thereby exploring the active role of animals in animal-assisted intervention. The low Cronbach's alpha value for interactions with objects may stem from the very low frequency of some behaviors (e.g., prosocial behaviors toward objects, which would only have been recorded if a child tried to “help” a toy, by cleaning or repairing it, or otherwise taking care of it). Repeating these analyses in future studies using control objects more likely to receive such attention from children (e.g., dolls or stuffed animals) will allow for further exploration of the internal reliability of this subscale. We currently recommend the use of subscales in the OHAIRE for interactions with animals, and the exploratory use of subscales for social interactions with peers, social interactions with adults, and interactions with objects. We recommend that researchers using these subscales present Cronbach's alphas in future publications for ongoing monitoring. We do not recommend using subscales for presenting behavior results in the behavioral categories of emotional display and problem behaviors). Additionally, while the current sample size did not lend itself to the use of factor analysis, future structure analyses for the OHAIRE may include factor analysis to confirm the suitability of the use of these subscales.

Finally, the OHAIRE has been used so far as a measure of behavior in studies of animal-assisted intervention including control groups where participants were not interacting with animals. Previously published results (11, 19) have shown its discriminative capacities, both between situations [e.g., children with ASD were found to smile more often in the presence of animals compared to toys, (11)], and between diagnostic groups [e.g., regardless of the situation, typically developing children smile more often than children with ASD; (39)]. Its use is apt to detect differences in the coded behaviors between situations with or without an animal. While it is not a diagnostic tool, the OHAIRE also shows sensitivity to behavioral differences between typically developing children and children with autism.

## Conclusion

The OHAIRE is a behavior coding tool that captures social interactions, emotional display, interfering behaviors, and interactions with animals and control objects. In the evaluated studies, the OHAIRE-v3 reached overall excellent levels of inter- and intra-rater reliability, limited correlations with caregiver-report questionnaires of social and interfering behaviors, and presents a reliable human-animal interaction subscale. Its current use is targeted to research teams aiming to examine and quantify children's behavior during animal-assisted intervention and continually monitor the psychometric properties of the coding tool. Its extension to new age ranges and diagnostic populations will evaluate its potential to have an even stronger impact in the field of HAI, as the first standardized behavior observation tool developed specially for human-animal interaction research.

## Author contributions

NG led the coding of study 1 for typically developing children and of studies 3 and 4, is a co-author of the behavior coding tool, and led reliability and validity analyses. RG was the principal investigator for studies 2 and 4, and is a co-author of the behavior coding tool. MG was an investigator on studies 2 and 4, and is a co-author of the behavior coding tool. RG and MG led behavior coding for study 2. SS was the principal investigator for study 3. AT and KT provided extensive statistical guidance for the reliability and validity analyses. SM and VS provided guidance in the initial development of the coding system and for study 1. MO developed the initial version of the OHAIRE behavior coding tool for study 1 and is its lead author, was the principal investigator for study 1, led the behavior coding for children with ASD in study 1, and provided extensive guidance for the behavior coding of all studies and for the reliability and validity analyses.

### Conflict of interest statement

The authors declare that the research was conducted in the absence of any commercial or financial relationships that could be construed as a potential conflict of interest.
